# The Role of mTOR Inhibitors for the Treatment of B-Cell Lymphomas

**DOI:** 10.1155/2012/435342

**Published:** 2011-06-16

**Authors:** Pinelopi Argyriou, Panagiota Economopoulou, Sotirios Papageorgiou

**Affiliations:** ^1^Department of Pathology, “Evangelismos” General Hospital of Athens, Athens, Greece; ^2^Haematology Unit, Second Department of Internal Medicine—Propaedeutic, University General Hospital “Attikon”, Greece; ^3^Department of Clinical Haematology, Royal Free Hospital, London NW3 5QG, UK

## Abstract

Despite the fact that the majority of lymphomas initially respond to treatment, many patients relapse and die from disease that is refractory to current regimens. The need for new treatment strategies in lymphomas has led to the investigation and evaluation of novel agents that target cellular pathways. The mammalian target of rapamycin (mTOR) is a representative pathway that may be implicated in lymphomagenesis. Rapamycin and especially its derivatives (temsirolimus, everolimus, and deforolimus) represent the first described mTOR inhibitors. These agents have shown promising results in the treatment of lymphoid malignancies. On the other hand, new ATP-competitive mTOR inhibitors that provoke a broader inhibition of mTOR activity are in early stages of clinical development. The purpose of this paper is to summarize the existing knowledge about mTOR inhibitors and their use in the treatment of B-cell lymphomas. Relevant issues regarding mTOR biology in general as well as in B-cell lymphoid neoplasms are also discussed in short.

## 1. Introduction

Current approaches in treating lymphoid malignancies have focused on the development of therapeutic regimens that selectively target dysregulated signal transduction pathways in neoplastic cells. Among aberrantly activated signaling cascades that are implicated in the pathogenesis of lymphomas is the mammalian target of rapamycin (mTOR) pathway, which is involved in many vital cellular processes [1]. Rapamycin and its analogs (rapalogs) comprise the classical mTOR inhibitors. A number of completed as well as other ongoing preclinical and clinical trials have tested these drugs in lymphomas, either as monotherapy or in combination with established chemotherapy [1]. Moreover, other anti-mTOR molecules, such as specific active-site TOR inhibitors (asTORi), with better pharmacological profiles are candidate drugs to be tested in clinical trials against lymphoid malignancies [2].

Herein we aim to review the results of trials with mTOR inhibitors in B-cell lymphomas. Firstly, the mTOR signaling network as well as possible aetiologic factors of aberrant activation of the mTORC1 signaling cascade in B-cell lymphoid malignancies are discussed in short.

## 2. mTOR Signaling Network

Rapamycin (also known as sirolimus or Rapamune, Wyeth) is the first described mTOR inhibitor [3]. This drug, originally developed as an antifungal agent, was soon found to have immunosuppressive and antineoplasmatic actions [4]. Systemic efforts to decipher the molecular mechanisms of these actions led to the isolation of the mTOR protein and the identification of two multimolecular complexes that are formed by mTOR, namely, the mTOR complex 1 (mTORC1) and 2 (mTORC2) [4, 5]. mTOR is the mammalian ortholog of a yeast serine-threonine kinase called target of rapamycin (TOR) [6]. Except for mTOR itself and the proteins mLST8/G*β*L [mammalian LST8 (lethal with SEC13 protein 8), also known as G protein beta subunit-like] and DEPTOR, which are common in both mTORC1 and mTORC2, several different constitutional proteins associate to form the two mTOR complexes [7, 8]. These multipeptidic structures are situated inside a signaling network, the mTOR network, characterized by many feedback loops and crosslinks among its various components [9].

Activity of mTORC1 is regulated by multiple molecular pathways that conduct input generated by growth factors, hormones, cytokines, amino acids, energy, stress- and oxygen-related signals [10–13]. Among these cascades are the PI3K/Akt (Phosphoinositide 3-kinase/Akt) and Raf/MEK/ERK (Raf/MEK/extracellular signal-regulated kinase) pathways, which are commonly activated in cancer and may cooperate in malignant transformation [9, 12]. Both pathways trigger the activity of mTORC1 through downregulation of the inhibitory effect of the TSC1-TSC2 (tuberous sclerosis complex 1-tuberous sclerosis complex 2) complex on Rheb (Ras homolog enriched in brain) protein [10, 12]. Akt kinase affects mTOR by way of two mechanisms. First, it lies upstream of mTORC1 and controls its activation. Second, Akt lies downstream of mTORC2 and depends on the latter as well as on PDK1 (pyruvate dehydrogenase kinase, isozyme 1) for full activation [7, 14].

Regulation of the two mTOR complexes bears some resemblance. For example, similarly to mTORC1, mTORC2 may also be activated by growth factors, hormones and amino acids, and this upregulation may be PI3K mediated [7, 15, 16]. In contrast, the TSC1-TSC2 complex, which suppresses mTORC1 activity, may promote mTORC2 signaling [10]. mTORC2 regulates Akt, SGK1(serum- and glucocorticoid-induced protein kinase 1), and PKC*α* (protein kinase C, alpha) phosphorylation and controls organization of actin cytoskeleton as well as cell size, cell cycle progression, proliferation, and survival [7, 15, 16]. The best characterized targets of mTORC1 are the S6 kinases [S6K1 (also known as p70S6) and S6K2] and the eukaryotic initiating factor-4e (eIF4e) binding proteins 1 and 2 (4E-BP1 and 4E-BP2) [9–11]. Upon activation, mTORC1 triggers vital anabolic processes such as ribosome biogenesis, cap-dependent translation, uptake of nutrients including glucose and amino acids, biosynthesis of amino acids, proteins, and lipids as well as (adenosine triphosphate) ATP sensing. Moreover, gene transcription, cell growth, cell cycle progression, proliferation, and survival are induced [4–7, 9, 17]. In addition, active mTORC1 downregulates macroautophagy and other catabolic processes such as fatty acid oxidation and protein degradation, while, in contrast, it stimulates aerobic glycolysis [4, 5, 17, 18].

Dysregulated activation of the mTORC1 pathway has been associated with tumor biology. Aberrant mTORC1 signaling disrupts homeostatic cell balance and contributes to uncontrolled proliferation and cell growth, survival, as well as angiogenesis and metastasis [9]. The same malignancy-inducing processes may be also promoted by abnormally elevated mTORC2 signaling [16, 19–21].

## 3. Aberrant mTORC1 Pathway Activation in B-Cell Lymphomas

Several lines of evidence indicate that aberrant activation of the mTORC1 pathway is common in both Hodgkin (HLs) and many types of B-cell non-Hodgkin lymphomas (NHLs) (Table 1) [22–25, 27, 28, 30–33, 40–42, 46–49]. However, the cause of this upregulation is currently poorly defined. Molecular events that affect signaling pathways related to mTORC1 complex modulation may presumably have an impact on the mTORC1 pathway itself [9]. Notably, the PI3K/Akt pathway, which is abnormally activated in many types of B-cell lymphomas, seems to participate in mTORC1 upregulation at least in a subset of these entities [22, 25, 27–29, 31–39, 41, 43–45, 47–49] (Table 1).

Several possible mechanisms of PI3K/Akt pathway activation in mature malignant lymphoid B cells have been described. These include (a) overexpression of membrane receptors which may be mutation related, and/or stimulation by their ligands by autocrine/paracrine secretion [23, 36–39, 41, 50], (b) aberrant tyrosine kinase (TK) activity [51], (c) constitution of oncogenic singalosomes [52], (d) expression of Epstein Barr virus (EBV) latent membrane protein 2A (LMP2A), and high levels of activated Ras protein [53, 54], (e) expression of the K1 protein of Kaposi's sarcoma-associated herpesvirus [55], (f) overexpression of the phosphodiesterase PDE4B gene and protein [56, 57], (g) overexpression of the T-cell leukemia/lymphoma 1 (TCL1) oncoprotein [58], (h) point mutations or amplification of the PI3K catalytic subunit alpha (PIK3CA) gene [29], and (i) genetic or epigenetic downregulation of phosphatase and tensin homolog (PTEN) suppressor gene [34, 35, 59, 60]. As for the latter, a very recent study in animal models, showed that PTEN cooperates with another negative modulator of PI3K-mediated signaling, the Src homology 2 domain-containing inositol phosphatase (SHIP), in order to suppress lymphoma pathogenesis [61].

**Figure 1:**
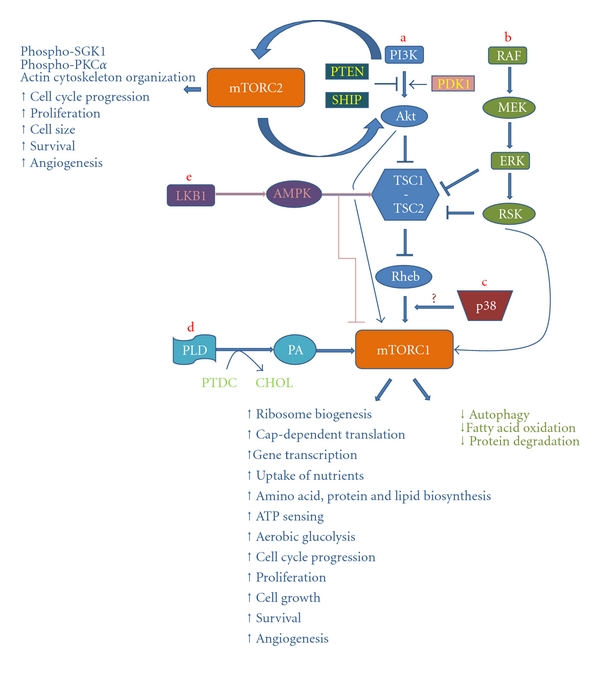
Molecular signaling cascades which normally control mTORC1 activity and may become dysregulated in B-cell lymphomas leading to aberrant mTORC1 signaling activation. The figure also demonstrates the functions of mTORC1 and mTORC2. (a) PI3K/Akt pathway: upon activation PI3K most possibly induces mTORC2 complex stimulation and also promotes the translocation of Akt and PDK1 to the cell membrane, where Akt becomes activated by PDK1 and mTORC2. Then, Akt activates mTORC1 by way of two mechanisms: (1) indirectly through downregulation of the inhibitory effect of the TSC1-TSC2 complex on Rheb protein and (2) directly through phosphorylation of PRAS40 (proline-rich Akt substrate of 40 kilodaltons), which is a component of the mTORC1 complex. The tumor suppressor phosphatases PTEN and SHIP oppose PI3K-mediated Akt activation. (b) RAF/MEK/ERK pathway: once activated this pathway triggers mTORC1 activity indirectly through inactivation of the TSC1-TSC2 complex by ERK and RSK (ribosomal S6 kinase, 90 kDa). The RAF/MEK/ERK pathway also directly activates mTORC1 through excitatory phosphorylation of raptor, a component of the mTORC1 complex, by RSK. (c) p38 is suggested to induce mTORC1 activity by acting downstream of or in parallel to Rheb. (d) PLD/phosphatidic acid (PA) pathway: upon activation PLD hydrolyzes phosphatidylcholine (PTDC) to generate choline (CHOL) and PA. Subsequently, PA activates mTORC1 by an unknown mechanism. (e) LKB1/AMP-dependent protein kinase (AMPK) pathway: the tumor suppressor kinase LKB1 activates AMP-dependent protein kinase (AMPK). AMPK, in turn, inhibits mTORC1 through activation of the TSC1-TSC2 complex and direct inhibitory phosphorylation of raptor.

Molecular alterations that activate the PI3K/Akt pathway could explain in part the upregulation of mTORC1 signaling in B-cell lymphomas (Figure 1(a)). The Raf/MEK/ERK pathway is another candidate inducer of mTORC1 activity in HLs and NHLs (Figure 1(b)). The fact that, on one hand, this pathway is another major upstream effector of mTORC1 and, on the other hand, it is activated in a subset of B-cell lymphomas justifies this hypothesis [24–26, 31, 38, 39, 42, 45] (Table 1). Indeed, there is evidence that upregulated Raf/MEK/ERK pathway may contribute to elevated mTORC1 signaling in the setting of follicular lymphoma (FL) and HL [25, 40, 42]. Apart from Erk, p38 is another mitogen-activated protein (MAP) kinase which was recently suggested to induce mTORC1 activity [13] and that may also become dysregulated in lymphomas [25, 62–64] (Figure 1(c)). Moreover, there is evidence for a role of the activated TK Syk in the upregulation of mTORC1 activity in FL, diffuse large B-cell lymphoma (DLBCL), mantle cell lymphoma (MCL), and Burkitt lymphoma (BL) cells. Notably, Syk-induced mTORC1 activation in FL cells appears not to be PI3K/Akt dependent [40]. Furthermore, Syk gene amplification and elevated protein expression was found in Jeko-1 MCL cell line and a few MCL tissue samples. These alterations could potentially be related to the activation of Syk protein and mTORC1 [65]. In addition, phospholipase D (PLD) seems to mediate mTORC1 stimulation in two FL cell lines, while its possible implication in mTORC1 activation in other lymphomas deserves further investigation [40] (Figure 1(d)). There are also data suggesting the contribution of serine/threonine kinase 11 (LKB1), a tumor suppressor kinase which negatively regulates mTORC1 activity, in lymphoma pathogenesis in animal models. However, whether LKB1 participates in human lymphoid malignancy induction remains uncertain at present [66] (Figure 1(e)). On the other hand, in a study in B-cell lymphoma cell lines mTORC1 upregulation was shown to be dependent on nutrients but not on other known upstream effectors [46]. As regards more proximal effectors of mTORC1, elevated levels of Rheb mRNA were found in some aggressive NHLs through an unknown mechanism and in individual cases of high increase were related to mTORC1 activation [67]. Finally, amplification of the RPS6KB1 gene, which encodes for p70S6/p85S6 protein, has been described in one third of a series of DLBCLs with unknown functional significance in mTORC1 signaling [33].

## 4. mTOR Inhibitors

### 4.1. Rapamycin and Rapalogs

The prototype of classical mTOR inhibitors is sirolimus [14]. The mechanism of action of sirolimus is rather complicated since it may inhibit mTORC1 or both the two mTOR complexes and either increase or in reduce Akt phosphorylation. These pharmaceutical effects are dependent on dose concentration, time after administration, and cell type [68–70]. Treatment with sirolimus may also activate ERK. Furthermore, sirolimus differentially affects the major substrates of mTORC1, S6K1 and 4E-BP1. It seems to downregulate S6K1, in most cell types, while in contrast inhibition of 4E-BP1 does not last long after treatment and is also cell specific. Consequently, a recovery in cap-dependent translation may be induced [17]. In malignant B-cells, sirolimus may cause cell cycle arrest, reduce proliferation, and inhibit growth in culture or delay tumor progression in animal models [47, 49, 70–72]. In addition, it may act similarly to amino acid deficiency as a positive modulator of genes which regulate nutrient catabolism and energy production and as a negative modulator of genes involved in the anabolic procedures of proteins, lipids, and nucleotides [73]. Moreover, it may potentiate the *in vitro* cytotoxicity of the chemotherapeutic agent doxorubicin, and the histone deacetylase inhibitor LBH [22, 70]. Of interest, sirolimus exhibits immunosuppressant properties and has been widely administered in patients with organ transplantation [3]. In addition, it may induce autophagy, both when given as monotherapy or in combination to radiation or dexamethasone [74–76]. 

Second generation rapamycin derivatives (rapalogs) with more favorable pharmacokinetic properties than the parent molecule, facilitating their clinical use, have been developed [77]. Currently, three of these chemical agents are available for clinical trials: temsirolimus (CCI-779, Torisel, Wyeth Pharmaceuticals), everolimus (RAD001, Affinitor, Novartis Pharmaceuticals), and ridaforolimus (AP23573, ARIAD Pharmaceuticals, formerly deforolimus) [78]. Similarly to rapamycin, rapalogs inhibit mTORC1, may downregulate mTORC2, and exert either excitatory or inhibitory effects on Akt protein, both *in vitro* and *in vivo*. Moreover, these effects depend on tumor-specific characteristics, dose, and schedule of treatment [70, 79, 80]. In B-cell lymphomas rapalogs exhibit antiproliferative, cytostatic, and antiangiogenic properties and may also trigger autophagy. In contrast, they appear to have minimal or no effect on survival of malignant cells [70, 81, 82]. Despite many theoretical gaps concerning rapalogs' mechanism of action, clinical trials with these agents show promising results in lymphoid neoplasms, apparently at the appropriate molecular background.

The pharmacokinetic and pharmacodynamic profiles of rapalogs differ. Temsirolimus is available in oral and intravenous formulations. Upon administration, it is rapidly converted to rapamycin, its primary active metabolite [83]. Phase I dose-finding studies for temsirolimus aimed to establish a maximum-tolerated dose through dose escalation, by testing either a schedule of a daily administration of 0.75 mg/m^2^ IV every other week with a 20% dose escalation or a weekly schedule of doses ranging from 7.5 mg–220 mg/m^2^IV [83, 84]. Maximum tolerated dose was not established but the maximum acceptable dose was 19 mg/m^2^/day due to grade 3 stomatitis. Although temsirolimus was found to be well tolerated, the most common toxicities included neutropenia, thrombocytopenia, asthenia, diarrhea, and stomatitis. The severity of its adverse effects was dose related. Because drug activity ceased to increase after several dose levels, phase I studies supported the use of a flat dose for temsirolimus and the suggested dose for phase II studies was 25, 75, or 250 mg weekly.

Everolimus is orally available and typically administrated on a daily schedule. It has been also been tested on a weekly basis, mostly in combination regimens. Phase I studies showed that the efficacy of the drug was dose dependent and that mTOR inhibition was more profound with daily dosing. In addition, everolimus was found to have acceptable tolerability at the highest doses studied. The suggested dose for phase II studies was 10 mg daily or 50–70 mg weekly [85, 86]. 

Ridaforolimus, unlike temsirolimus, is not a prodrug [87], and it is given typically intravenously, although oral administration is currently under clinical testing [88]. Phase I studies tested a daily regimen of 3–28 mg and found that mTOR inhibition increased in a less than proportional manner. Maximum tolerated dose was 18.75 mg daily due to grade 3 mouth sores and a dose of 12 mg IV daily was proposed as suitable for phase II studies [87].

At present, numerous clinical trials are under way in order to evaluate the above drugs as single agents or in combination in aggressive and/or refractory lymphomas (Table 2) (http://www.cancer.gov/search/ResultsClinicalTrials).

## 5. Clinical Trials with Rapalogs in Lymphomas

### 5.1. Mantle Cell Lymphoma

Mantle Cell Lymphoma (MCL) is an aggressive type of mature B-cell lymphoid neoplasm with a relative frequency of 7% among NHLs. The genetic hallmark of MCL is the translocation t(11;14)(q13;q32), which results in overexpression of cyclin D1 [89]. It is characterized by an aggressive clinical course and poor prognosis with median survival of 3 to 5 years [90]. Although front treatment induces a high rate of complete remission (CR), relapse is common. Therefore, new therapies are needed [91]. Aberrant activation of mTORC1 as well as PI3K/Akt signaling is frequent in the MCL [27, 28, 30, 49].

Additionally, in preclinical MCL models both temsirolimus and everolimus showed anti-proliferative effects, especially in combination with other therapeutic agents, such as vorinostat, doxorubicin, and vortezomib [81, 82, 92, 93].

Among rapalogs, temsirolimus has been thoroughly studied in clinical trials in MCL. In two phase II studies and in one large randomized phase III study performed by North Central Cancer Treatment Group (NCCTG), temsirolimus was found to display significant antitumor activity and clinical benefit as a single therapeutic agent in relapsed or refractory MCL. The first of phase II studies conducted by Witzig et al., assessed the efficacy of a 250 mg/week IV course of temsirolimus monotherapy in 35 patients with relapsed or refractory MCL that had received previous treatment. The overall response rate (ORR) was 38%, with one complete remission (CR) and one partial remission (PR) [94]. In the second phase II study, Ansell et al. administered a 10-fold lower dose of temsirolimus (25 mg weekly IV) in 29 patients with relapsed or refractory MCL and achieved a similar ORR (41%) with one CR and ten PRs. However, the lower dose was associated with lower rates of toxicity (50% versus 71% grade 3 and 4% versus 11% grade 4) [95]. In both studies, thrombocytopenia was the most common adverse effect and the most frequent cause of dose reduction. In addition, both studies included adults (median age 70 years old) that had failed previous therapies with rituximab (monoclonal anti-CD20 antibody), cyclophosphamide, or doxorubicin [94, 95].

Based on these results, a randomized, large phase III study was conducted to evaluate the effect of temsirolimus in comparison to investigator's choice therapy in 162 patients with relapsed/refractory MCL, previously treated with rituximab, alkylating agents, and anthracycline. The patients were randomized to receive treatment with temsirolimus applied in one of two therapeutic schemes (175 mg/week for three weeks followed by either 25 mg or 75 mg weekly IV) or treatment with a single agent of the investigator's choice from approved protocols. It was shown that ORR was significantly higher in patients who received the 75 mg dose of temsirolimus compared to treatment with the investigator's choice agent (22% versus 2%, *P* = .0019). Median progression free survival (PFS) was also longer (4.8 months versus 1.9 months). Regarding patients who received the of 25 mg temsirolimus, ORR was 6% and PFS 3.4 months. Similar to the previous trials, hematological toxicity was the most frequent adverse effect. This study demonstrated that administration of 175/75 mg temsirolimus improved ORR and PFS significantly and showed a trend toward longer overall survival (OS) [96]. The results of this trial led to the European approval of temsirolimus as single agent therapy for the treatment of relapsed/refractory MCL [97].

 More recently, Ansell et al. reported the results of the first phase II study that examined the efficacy of temsirolimus in combination with rituximab in patients with relapsed or refractory MCL [98]. In this study 69 patients were treated with temsirolimus (25 mg/week) and rituximab (375 mg/m^2^ per week) for 4 weeks during the first cycle followed by a single dose of rituximab every other 28-day cycle for a total of 12 cycles. The ORR was 59% consisting of 19% CRs and 41% PRs. The ORR was 63% for rituximab-sensitive patients and 52% for rituximab-refractory patients. The median time to progression (TTP) for all patients was 9.7 months (10.9 months in the rituximab-sensitive patients and 5.4 months in the rituximab-refractory patients). The most common side-effect was hematological toxicity which did not differ from that in the previous studies of temsirolimus alone. Additionally, the other more frequent grades 3 and 4 toxicities included increased serum concentrations of cholesterol and triglycerides, hyperglycaemia, fatigue, and dyspnoea. The frequencies of these toxicities were also similar to that of temsirolimus as monotherapy suggesting that rituximab can be safely combined with temsirolimus without much increase in toxicity. The above results are promising with much higher ORR and CR rate than in the phase III study of temsirolimus alone without increasing toxicity [96]. However, more randomized trials are needed in order to establish the effectiveness of the combination of temsirolimus plus rituximab in the treatment of relapsed or refractory MCL patients. 

The efficacy of everolimus in MCL was investigated in phase II clinical trials. Witzig et al. demonstrated the antitumor activity of the drug when applied as monotherapy in relapsed/refractory NHLs. In this trial, 19 patients with MCL, 47 patients with DLBCL and 3 patients with FL were included. Daily dose was 10 mg PO. All patients had been heavily pretreated with a median of three previous therapies and 32% of them had undergone autologous stem-cell transplantation. In this study, ORR in patients with MCL was 32%, lying in the middle of the 40% and 22% that were found in the two phase II and the one phase III trials of temsirolimus, respectively. In addition, ORR in DLBCL patients was 30% and in FL patients 38%. Hematological toxicity, mainly thrombocytopenia (38%), was again the most frequent adverse effect. Grade 3 or 4 toxicity was observed in 68% of the patients, which was managed easily with dose interruption or reduction [99]. On the other hand, in another phase I/II study designed to evaluate everolimus effect in 26 patients with hematological malignancies including MCL, none of the 4 patients with MCL responded to everolimus [93].

Furthermore, in the setting of MCL the activity of ridaforolimus has been evaluated as well. In a phase II study, 55 heavily pretreated patients with various hematological malignancies, including 9 patients with MCL, received ridaforolimus as single agent (12.5 mg IV once a week every 2 weeks). The most favorable response was observed in MCL patients, with 33% ORR and three PRs. Although the number of MCL patients involved in this study was small, it cannot be ignored that ORR achieved with ridaforolimus is similar to that demonstrated in the two phase II studies of single agent temsirolimus in MCL. Additionally, the fact that 44% of MCL patients had stable disease and only two experienced progressive disease probably reflects a promising antitumor activity of ridaforolimus that has to be further investigated. It is also noteworthy that ridaforolimus was well tolerated. Mouth sores was the most frequent adverse effect, while thrombocytopenia was less commonly encountered than with other rapamycin derivatives [100].

In summary, temsirolimus is the most extensively studied mTOR inhibitor in the setting of MCL, which has been shown to significantly improve objective response and progression-free survival compared to investigator's choice therapy in patients with relapsed/refractory MCL in a phase III clinical trial. The effectiveness of temsirolimus in combination to immunotherapy or chemotherapy has already been under investigation. Everolimus and ridaforolimus have demonstrated promising antitumor activity against MCL but further investigation is needed in order to evaluate their potential efficacy.

### 5.2. Diffuse Large B-Cell Lymphoma (DLBCL)

Diffuse large B-cell lymphoma (DLBCL) represents almost one third of all NHL subtypes [90]. Although standard chemotherapy regimens (R-CHOP, rituximab-cyclophospamide, doxorubicin, vincristine, and prednisone) have shown effectiveness in the treatment of DLBCL, there is still a group of patients that die from the disease [101].

In preclinical studies, rapamycin analogue everolimus has been found to induce G1 cell-cycle arrest but not apoptosis in DLBCL cell lines of the germinal centre (GC) type and to increase the cytotoxicity of rituximab [71, 102].

Temsirolimus has shown promising results as a single agent in DLBCL in a phase II study performed by University of Chicago. The study included 89 pretreated patients with either DLBCL, follicular lymphoma (FL), chronic lymphocytic leukemia (CLL), or other indolent lymphomas that were stratified in three groups. Patients received a weekly course of temsirolimus of 25 mg. It was found that DLBCL patients had an ORR of 28.1% with four CRs and 5 PRs (9 patients out of 32). This result is promising, taking into account that all patients were heavily pretreated; however, the durability of response was short (2.4 months). Based on the fact that nearly half of DLBCL responders had transformed from a prior FL, authors suggest that temsirolimus might be more active in follicle center derived lymphomas. The most common adverse effect of temsirolimus was myelosuppression, which was reversible, while other toxicities included stomatitis and metabolic dysregulation mainly grade one or two [103].

Everolimus has also been tested in clinical trials in DLBCL, demonstrating response rates similar to temsirolimus. In a phase II study by Witzig et al. that included 77 patients with DLBCL, FL, or MCL, the ORR in DLBCL patients was 30% (14 out of 47) [99]. However, the duration of response was longer than with temsirolimus (5.7 months). On the other hand, compared to the previous study with temsirolimus where 4 CRs were observed, no patient among DLBCL group who were treated with everolimus achieved a CR. The most important adverse effect in this study was grade 3 or 4 hematologic toxicity, which appeared in 68% of patients [103].

Deferolimus has not been investigated yet as a single agent in the treatment of DLBCL or other NHL subtypes. However, preliminary results show antitumor activity in many tumor types and numerous ongoing clinical trials are under way (Table 2) [87].

### 5.3. Follicular Lymphoma (FL)

Follicular lymphoma (FL) is the second most common type of B-NHL in the West, accounting for 25–35% of all NHLs [90]. Patients with FL usually have an indolent clinical course, but they might eventually evolve to a refractory phase that can lead to death. Activation of mTOR pathway has been demonstrated in FL cell lines and tissue samples [40–42]. It has also been shown that mTOR activation in FL cell lines is enhanced by Syk independently of Akt and also by PLD [40]. However, mTOR inhibitors have not been tested in FL cell lines or animal models.

Both temsirolimus and everolimus have shown effectiveness in relapsed FL in phase II studies. In the above mentioned study from University of Chicago, patients with FL demonstrated an ORR of 53.8% with CR rates reaching 25.6%. Furthermore, median duration of response was 13 months, much longer than in DLBCL [103]. Similarly to temsirolimus, everolimus has shown antitumor activity in FL. In the previously mentioned phase II study by Witzig et al. the reported ORR in FL was 38%, with a median duration of response of 5.7 months. Of note, this study included only patients with FL grade 3 [99]. These results are very promising, but they need to be validated in further studies that will include larger groups of patients.

### 5.4. Hodgkin's Lymphoma (HL)

Hodgkin's Lymphoma (HL) represents 30% of all lymphoma cases. It is characterized by the presence of neoplastic Hodgkin and Reed-Steinberg cells (HRS) in a background of inflammatory and accessory cells [90]. Although it has proven a highly curable disease, there is a subset of patients that either relapse after salvage chemotherapy or do not respond due to old age. Consequently, there is still need for new therapeutic approaches.

 Various studies have demonstrated activation of the mTOR pathway in HL cell lines and primary tumors [104–107]. The mechanism of mTOR activation in HL has not been clarified yet, but Akt is considered to play a role since phosporylated Akt has been reported to be activated in HL tumors [22, 108]. In HRS cells, rapamycin induces G1-S cell cycle arrest but not apoptosis and enhances the cytotoxic activity of doxorubicin [109]. Among rapamycin analogues, temsirolimus has been reported to induce cell cycle arrest followed by autophagy in HL cell lines [110]. In addition, everolimus has been demonstrated to have antiproliferative results in HRS cells. Furthermore, it has been shown to be effective in HL murine xenograft models [109].

Based on preclinical studies, a phase II study assessed the effectiveness of everolimus in patients with refractory/relapsed HL. This study evaluated a total of 19 patients with relapsed/refractory HL as part of a larger study evaluating everolimus in more rare forms of lymphoma. Patients had a median age of 37 years and had received a median of six prior therapies. They were treated with a daily oral dose of 10 mg and response was evaluated after two and six cycles of therapy. The ORR was 47%, with 8 PRs and one CR. Among those patients, 4 remained progression free at 12 years and 1 remains on therapy for more than 3 years. Of note, 74% patients experienced grade 3 or 4 toxicity. Although hematological toxicity was the most common adverse effect, a subset of patients (11%) developed pulmonary symptoms, such as dyspnea and cough that required dose reductions. This high rate of toxicity should be a matter of concern in older patients. Overall, the results of this study are encouraging, if we take into account that responders had stable disease on everolimus for a long period. The authors suggest that combination of everolimus with other agents might be even more effective [110].

## 6. Specific and NonSpecific ATP-Competitive mTOR Inhibitors

Recently a new category of mTOR inhibitors has come to prominence due to their ability to show a more profound impact on PI3K/Akt/mTOR pathway in relation to rapamycin and rapalogs. These drugs are small molecules that bind to the ATP-binding site of mTOR kinase and inhibit the catalytic activity of both mTOR complexes [2, 111–122]. Two subclasses of agents are included here. The first subclass comprises of nonspecific ATP-competitive mTOR inhibitors, which apart from mTORC1 and mTORC2 also inhibit PI3Ks (Dual PI3K/mTOR inhibitors) [112–116]. The second subclass consists of drugs which selectively inhibit mTORC1 and mTORC2 without affecting other kinases [111, 117–122]. These molecules are known with different names such as specific active-site TOR inhibitors (asTORi) and TOR kinase domain inhibitors (TORKinibs) [2, 115, 123].

## 7. Dual PI3K/mTOR Inhibitors

This subclass of ATP-competitive mTOR inhibitors includes several molecules which have been applied in preclinical models in hematologic malignancies [2, 27, 28, 49, 102, 116, 124, 125]. Two of these agents, LY294002 and wortmannin, were initially described as PI3K inhibitors and later found to target mTOR as well [124]. With regard to B-cell lymphomas, both of them have been tried in MCL and FL cell lines and were shown to downregulate Akt and/or mTOR activity [27, 28, 40, 49]. In addition, LY294002 was shown to decrease cyclin D1 protein levels in MCL cells, suggesting induction of cell cycle arrest [27]. LY294002 was also applied on DLBCL cell lines and found to trigger apoptosis in 3 out of 5 cell lines in one study. In the same study two DLBCL cell lines excibited dephosphorylation of Akt upon LY294002 treatment [102]. Similar were the results regarding LY294002 effect on Akt inactivation in another study from China which also included DLBCL cell lines. Moreover, in this study, LY294002 decreased the ratio of S phase and of interest exhibited synergistic effect with doxorubicin on triggering apoptosis [126]. Another dual PI3K/mTOR inhibitor, NVP-BEZ235 was recently tried in FL cell lines and was found to inhibit cell growth and proliferation due to increased apoptosis. Furthermore, it showed a synergistic activity with bortezomib against FL cell proliferation [116]. Furthermore, Bhatt et al. reported that NVP-BEZ235 suppressed proliferation in primary effusion lymphoma (PEL) cell lines and xenograft models, more efficiently than selective inhibitors of PI3K/Akt mTOR pathway [48]. Although results from preclinical trials with dual PI3K/mTOR inhibitors are preliminary and further clinical trials are needed to confirm them, these agents seem that may be potentially effective in NHL treatment. Dual PI3K/mTOR inhibitors are currently being tested in phase I trials [112].

## 8. Active-Site TOR Inhibitors (asTORi)

All preclinical compounds in this category have been reported to have similar molecular behaviour, as they have been shown to reduce phosphorylation of both endogenous S6 kinase and Akt [115, 117–119, 122]. Interestingly, they are more effective mTORC1 inhibitors than rapamycin and rapalogs, since they completely inhibit S6 kinase and 4EBP1. Also, they trigger a more intense suppression on cap-dependent translation, protein synthesis, cell growth and proliferation [2, 115, 117–119, 122]. In addition, asTORi may induce apoptosis and autophagy and in relation to rapalogs cause a more profound decrease of lactate as well as the angiogenic hypoxia inducible factors (HIFs) and vascular endothelial growth factor (VEGF) [121, 122]. Among asTORi agents used in clinical models, AZD8055 shows similar *in vitro* effects with PP42, Torin-1, and Ku-0063794. However, in contrast to these inhibitors AZD8055 has also been found to inhibit tumor cell proliferation *in vivo* [121]. More specifically, it induces a dose-dependent inhibition and/or regression in human tumor xenograft models which is associated with a dose-dependent pharmacodynamic effect on both phosphorylated S6 and phosphorylated Akt. AZD8055 is currently being evaluated in phase I studies [122]. PP42 and Ku-0063794 are two other asTORi drugs which have shown important preclinical activity against hematological malignancies [2]. PP42 has been found to cause death to mouse and human Ph+ B-ALL cells, with great selectivity to leukemia cells compared to normal bone marrow and peripheral blood lymphocytes [120]. Moreover, PP42 has shown marked therapeutic response in transgenic mice that develop thymic lymphomas [127]. Regarding another asTORi, OSI-027, it has been reported to generate antileukemic effects in BCR/ABL transformed cells. Based on this finding, it is currently being evaluated in phase I studies in solid as well as lymphoid neoplasms. Finally, INK128 has demonstrated broad preclinical antitumor activity against a range of solid tumor types and multiple myeloma [2]. Currently, it is also being evaluated in phase I studies. None of these inhibitors have been tested in clinical trials in lymphomas yet. Further studies are currently conducted aiming to elucidate the potential therapeutic effect of asTORi-s in neoplasms, including lymphomas.

## 9. Conclusions

Through the last years it has become clear that mTOR pathway contributes to the pathogenesis of hematological malignancies by playing a key role in the regulation of many cell functions, such as cell proliferation, cell growth, and angiogenesis. The development of mTOR inhibitors has opened a new field in the clinical arena of lymphomas. Temsirolimus has been recently approved for the treatment of relapsed/refractory mantle cell lymphoma. In addition, everolimus and deferolimus have been evaluated in phase II clinical trials that reveal their potential for the treatment of aggressive lymphomas, justifying further evaluation with randomized phase III trials. In addition to rapalogs, other types of mTOR inhibitors have been currently developed, with promising results in preclinical studies. In our opinion, future research on the use of mTOR inhibitors in lymphoid malignancies should aim in three basic fields: (a) identification of the whole spectrum of molecular alterations that are related to mTOR signaling dysregulation in each lymphoma subtype, (b) definition of the subset of patients who are likely to respond best to anti-mTOR treatment, and (c) design of new clinical trials aiming to determine the effectiveness of mTOR-inhibitors in the context of established or other; targeted or nontargeted; treatment. Undoubtedly, there is evidence-based hope that lymphoma treatment will be substantially improved in the next decade.

## Figures and Tables

**Table 1:** Evidence of aberrant activation of mTORC1, PI3K/Akt, and Raf/MEK/ERK pathways in B-cell lymphomas.

Lymphoma Type	mTORC1 activation	PI3K/Akt activation	MEK/ERK dysregulation	Ref/s
Hodgkin Lymphoma (HL)	Cell lines, tissue samples	Cell lines, tissue samples		[22]
	Tissue samples			[23, 24]
	Tissue samples	Tissue samples	Tissue samples	[25]
			Cell lines, tissue samples	[26]

Mantle Cell Lymphoma (MCL)	Cell lines, tissue samples	Cell lines, tissue samples		[27, 28]
	Cell lines	Cell lines, tissue samples		[28]
		Cell lines, tissue samples		[29]
	Tissue samples			[30]
	Cell lines, Lymphoma cells from a MCL patient	Cell lines, Lymphoma cells from a MCL patient	Cell lines, Lymphoma cells from a MCL patient	[31]

Diffuse Large B-Cell Lymphoma (DLBCL)	Tissue samples	Tissue samples		[32]
	Cell lines, tissue samples	Cell lines		[33]
		Tissue samples		[34, 35]
		Cell lines, tissue samples		[36, 37]
		Cell lines	Cell lines	[38, 39]

Follicular Lymphoma (FL)	Cell lines, tissue samples			[40]
	Cell lines		Cell lines	[41]
	Cell lines	Cell lines		[42]
		Cell lines		[43, 44]
		Cell lines	Cell lines	[45]

Burkitt Lymphoma	Cell lines			[24]
		Cell lines	Cell lines	[32, 46]

Primary Effusion Lymphoma (PEL)	Cell lines, animal model (mice)	Cell lines, animal model (mice)		[47, 48]
	Cell lines		Cell lines	[24]

**Table 2:** Ongoing clinical trials with rapalogs in patients with NHL.

Phase	Locations	Clinical trial gov. number	Additional information
I/II	USA	NCT01076543	Lenalidomide and Temsirolimus in patients with relapsed or refractory HL or NHL

I/II	USA	NCT00787969	Rituximab, Cladribe, and Temsirolimus in patients with newly diagnosed MCL

I/II	GERMANY	NCT01078142	Temsirolimus, Bendamustine, and Rituximab for relapsed FL or MCL (BERT)

I/II	USA	NCT00474929	Everolimus and sorafenib for relapsed or refractory NHL, HL, or MM

I	Cleveland OH	NCT00671112	Everolimus plus bortezomibe for relapsed/refractory MCL and other NHL

I/II	USA	NCT00967044	Panobinostat (LBH589) plus Everolimus (RAD001) in patients with relapsed and refractory Lymphoma

I/II	USA	NCT 101075321	Everolimus and Lenalidomide in treating patients with relapsed or refractory NHL or HL

I/II	USA	NCT00918333	Panobinostat and Everolimus in treating patients with recurrent MM, NHL, or HL

II	USA	NCT00869999	Everolimus plus Rituximab for relapsed/refractory DLBCL

I/II	USA	NCT00704054	Deferolimus for relapsed/refractory NHL/HL
